# Metabolic Profiling of the Response to an Oral Glucose Tolerance Test Detects Subtle Metabolic Changes

**DOI:** 10.1371/journal.pone.0004525

**Published:** 2009-02-26

**Authors:** Suzan Wopereis, Carina M. Rubingh, Marjan J. van Erk, Elwin R. Verheij, Trinette van Vliet, Nicole H. P. Cnubben, Age K. Smilde, Jan van der Greef, Ben van Ommen, Henk F. J. Hendriks

**Affiliations:** 1 Department Quality of Life, TNO, Zeist, the Netherlands; 2 Biosystems Data Analysis, Swammerdam Institute for Life Sciences, University of Amsterdam, Amsterdam, the Netherlands; Institute of Genomics and Integrative Biology, India

## Abstract

**Background:**

The prevalence of overweight is increasing globally and has become a serious health problem. Low-grade chronic inflammation in overweight subjects is thought to play an important role in disease development. Novel tools to understand these processes are needed. Metabolic profiling is one such tool that can provide novel insights into the impact of treatments on metabolism.

**Methodology:**

To study the metabolic changes induced by a mild anti-inflammatory drug intervention, plasma metabolic profiling was applied in overweight human volunteers with elevated levels of the inflammatory plasma marker C-reactive protein. Liquid and gas chromatography mass spectrometric methods were used to detect high and low abundant plasma metabolites both in fasted conditions and during an oral glucose tolerance test. This is based on the concept that the resilience of the system can be assessed after perturbing a homeostatic situation.

**Conclusions:**

Metabolic changes were subtle and were only detected using metabolic profiling in combination with an oral glucose tolerance test. The repeated measurements during the oral glucose tolerance test increased statistical power, but the metabolic perturbation also revealed metabolites that respond differentially to the oral glucose tolerance test. Specifically, multiple metabolic intermediates of the glutathione synthesis pathway showed time-dependent suppression in response to the glucose challenge test. The fact that this is an insulin sensitive pathway suggests that inflammatory modulation may alter insulin signaling in overweight men.

## Introduction

The low-grade inflammatory state often seen in overweight subjects is thought to play an important role in lifestyle associated disease development. This inflammatory state has been associated with cardiovascular diseases [1], diabetes, insulin resistance [2] and cancer [3]. Since the early 1990s [4], considerable effort has been made to discover and validate biomarkers with diagnostic or prognostic utility for lifestyle associated diseases [5], [6], [7]. Metabolites such as cholesterol, fasting glucose and homocysteine have long been used as biomarkers. Genomic – based technologies such as metabolic profiling provide a new means to explore the combination of multiple metabolites as a biomarker, which may allow for more precise outcome predictions. Alternatively, such a biomarker may provide a more comprehensive insight into pathophysiological processes not previously attainable with traditional biomarkers [8], [9], [10]. These markers should respond to nutritional and pharmaceutical interventions in order to be evaluated.

The main focus of the present study was to demonstrate and quantify the consequence of using diclofenac to reduce inflammation and its effect on metabolism. Subsequently, the study was geared to identify multiple metabolites to be used as a potential biomarker. Diclofenac acts as a non-selective inhibitor of the enzymes cyclooxygenase-1 and -2. Cyclooxygenases catalyze among other things the formation of prostaglandins that act as messenger molecules in inflammation. Metabolic profiling has been shown to be a valuable tool to quantify nutritional metabolic homeostasis and disease mechanisms associated with metabolic stress and metabolic syndrome [11], [12], [13]. Liquid and gas chromatography mass spectrometric methods (LC-MS and GC-MS) were used to detect high and low abundant metabolites in plasma to obtain a comprehensive picture of metabolic changes induced by a mild anti-inflammatory drug intervention. A total of 343 plasma metabolites were quantified, of which 204 could be identified, spanning diverse chemical classes (Table S1). The metabolic profiling approach was not only applied in fasting (homeostatic) conditions, but also at multiple time points during an oral glucose tolerance test (OGTT). This approach is based on the concept that the resilience of the system can be assessed after challenging or perturbing a homeostatic situation. Plasma metabolic profiling combined with a glucose challenge has already been successfully used to differentiate between healthy individuals and individuals with an impaired glucose tolerance [14]. Applying a metabolic perturbation and metabolic profiling may help identify a set of metabolites that predict differences in the responses between treatment groups to the oral glucose tolerance test. A similar set of metabolites might then provide novel insight into the interplay between metabolic and inflammatory processes and provide candidate biomarkers to be applied in (intervention) studies aimed at lifestyle associated diseases.

## Materials and Methods

### Ethics statement

The study was approved by the Medical Ethics Committee of the University Medical Centre of Utrecht (May 17, 2005). In total, fifty subjects gave written informed consent after being informed about the study, both verbally and in writing.

### Subjects and study design

The study was conducted at TNO Quality of Life (Zeist, the Netherlands). Overweight and mildly obese men (Body Mass Index (BMI) between 25.1 and 34.0 kg/m2) were recruited from a pool of volunteers. All fifty subjects completed a questionnaire on medical history and were physically examined. Blood and urine were collected after an overnight fast for routine analysis. In addition, plasma hsCRP levels were determined.

Subjects met the following in- and exclusion criteria. Smokers, subjects who reported that they were trying to lose weight or who were on a medically prescribed diet and subjects with allergy or hypersensitivity for non-steroidal anti-inflammatory drugs were excluded from participation. Additionally, subjects who were on medication that may have interfered with parameters to be measured or with diclofenac treatment or subjects who, based on anamnesis, were not suitable to receive diclofenac treatment (history of current gastro-intestinal diseases including bleeding, ulcer or perforation, history of stroke, history of current significant haematological disorders, any significant hepatic, renal or cardiovascular disease, asthma) or subjects with a history of medical or surgical events that may have affected the study outcomes were not included. Based on these criteria, 25 subjects were eligible. Of the 25 eligible subjects, the 5 subjects with the lowest CRP values were not included in the study. Levels of hsCRP of the included subjects ranged from 0.41–9.72 mg/L (see also Table 1).

**Table 1:** Demographic data of subjects that completed the study (n = 19) at screening; mean±SD (range).

	All (n = 19)	Placebo treatment (n = 10)	Diclofenac treatment (n = 9)
Age (years)	43±15	41±16 (19–60)	45±15(21–58)
Body weight (kg)	93.5±8.0	93.5±9.3 (81.1–105.2)	93.5±6.9 (85.2–104.4)
Height (m)	1.82±0.08	1.82±0.10(1.69–1.96)	1.83±0.07 (1.70–1.92)
BMI (kg/m^2^)	28.1±1.2	28.1±1.0 (26.7–29.3)	28.1±1.5 (26.1–30.9)
hs-CRP (mg/L)	2.22±2.33	2.08±1.88 (0.41–6.35)	2.37±2.87(0.64–9.72)
Fasting glucose (mmol/L)	6.0±0.5	5.9±0.5 (5.2–7.1)	6.0±0.6 (5.0–6.8)
Fasting insulin (mU/L)	13.4±8.1	13.4±8.6 (5.1–26.8)	13.3±8.1 (3.3–26.6)

The study was designed as a double blind, randomized, parallel trial, in which subjects were treated with diclofenac (n = 10) or placebo (n = 10). Randomization of subjects to treatment groups was restricted by hsCRP, body mass index (BMI), fasting glucose and age. The result is a homogeneous division of these parameters over the two treatment groups at the start of the study (see Table 1). Subjects consumed one capsule (placebo or 50 mg diclofenac) approximately one hour before breakfast, lunch and dinner for 9 days. Subjects were instructed to keep to their habitual diet during the study. One person dropped out on the first day of the study for study unrelated reasons. Nineteen men completed the study. Their subject characteristics are presented in Table 1. Prostaglandin E2 concentrations were used as a readout for diclofenac treatment and showed a significant reduction in subjects treated with diclofenac (p = 0.02). Prostaglandin E2 concentrations were unchanged in subjects treated with placebo demonstrating a modulation of the inflammatory status in diclofenac treated subjects (Table 2).

**Table 2:** Characteristics of prostaglandin E2 and OGTT parameters (insulin and glucose) measured at start and end of treatments. AUC = area under the curve.

	Placebo		Diclofenac	
	Day 0 Mean (st dev)	Day 9 Mean (st dev)	Day 0 Mean (st dev)	Day 9 Mean (st dev)
PGE2 [pg/mL]	56.5 (7.7)	60.8 (11.0)	55.3 (11.9)	48.9 (10.6)
AUC glucose [mmol*min/L]	409 (238)	306 (197)	365 (255)	262 (199)
AUC insulin [mU*min/L]	8986 (5356)	8935 (4821)	12140 (12037)	9965 (9078)

**Figure 1:**
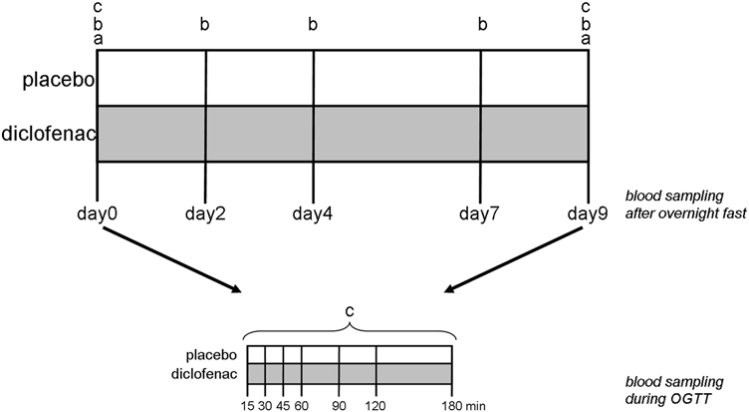
Overview of study design, time points at which metabolome was measured and multivariate data analyses. To determine metabolites that were modulated by the diclofenac treatment the following multivariate data comparisons were performed to identify metabolites that were modulated by the diclofenac treatment: a) PLS-DA on metabolic profiling data from day 9 subtracted by metabolic profiling data from day 0, on fasted plasma samples; b) n-PLS-DA on metabolic profiling data from day 0, 2, 4, 7 and 9, on fasted plasma samples; c) n-PLS-DA on metabolic profiling data from day 9 subtracted by metabolic profiling data from day 0, using the fasted plasma samples and the samples after glucose administration, thus metabolic profiling data on 0, 15, 30, 45, 60, 90, 120 and 180 minutes after glucose administration. The multivariate data comparisons from a-c were performed per metabolite platform, thus multivariate models were created for GC-MS global, LC-MS polar, LC-MS lipids and LC-MS free fatty acids data.

Blood samples were taken after an overnight fast on days 0, 2, 4, 7 and 9. Subjects underwent an oral glucose tolerance test (OGTT) on day 0 and day 9. Blood samples were taken just before (0 minutes) and 15, 30, 45, 60, 90, 120 and 180 minutes after the administration of the glucose solution (75 grams). Samples were analyzed for glucose and insulin for which the incremental area under the response curves (AUC) was calculated. Table 2 shows the characteristics of these parameters. No significant changes were observed between the treatments. Figure 1 shows study design and time points at which metabolic profiling measurements were done.

### Plasma PGE2, insulin and glucose measurements

PGE2 was determined using the Prostaglandin E2 [^125^I] Biotrak assay system (Amersham Biosciences, UK) with modifications. In short, PGE2 in samples was derivatized to the methyl oximate derivative. The resulting solution was further diluted (final dilution 5 times) in PBS and partly assayed. The assay consists of incubation of the oximated sample PGE2, the ^125^I-labelled PGE2, and a PGE2 specific antibody. After incubation, the Amerlex-M reagent is added and the free and bound ^125^I labeled PGE2 separated using centrifugation. The resulting bound radioactivity in the pellet is determined using a gamma-counter. Serum glucose concentrations were measured by using a commercial test kit (Roche diagnostics GmbH, Mannheim, Germany) on a Hitachi 911 automatic analyzer (Hitachi Instrument Division, Ibaraki-ken, Japan), with intra-assay CVs that ranged between 0.7% and 0.9%, depending on the concentration. Serum insulin concentrations were measured using an AIA-600 Immunoassay Analyzer (Tosoh Corporation, Toyama, Japan), with intraassay CVs that ranged between 4.3% and 5.8%, depending on the concentration.

### Metabolic profiling measurements

#### LC-MS of lipids and fatty acids

Plasma lipids and free fatty acids (FFA) were analyzed with electrospray LC-MS [15], [16]. The instrument used was a Thermo LTQ equipped with a Thermo Surveyor HPLC pump. Data were acquired by scanning the instrument form m/z 300 to 1200 at a scan rate of approximately 2 scans/s. The FFA LC-MS platform employs the same HPLC conditions as the lipid method except for the gradient. Detection of FFA is performed in negative ion mode, and lipids are measured in positive ion mode. Taken together the two methods can measure approximately 200 different identified lipids and FFA.

In summary, 10 µl of plasma was extracted with 300 µl of isopropanol containing several internal standards (IS: C17:0 lyso-phosphatidylcholine, di-C12:0 phosphatidylcholine, tri-C17:0 glycerol ester, C17:0 cholesterol ester and heptadecanoic acid (C17:0)). Each extract was injected three times (10 µl), once for the LC-MS FFA platform and two times for the LC-MS lipid platform. Furthermore, a quality control (QC) sample was prepared by pooling of plasma from all subjects. The pool was divided into 10 µl aliquots that were extracted the same as the study samples. The QC samples were placed at regular intervals in the analysis sequence (one QC after every 10 samples). The QC samples served two purposes. The first is a regular quality control sample to monitor the LC-MS response in time. After the response has been characterized, the QC samples were used as standards of unknown composition to calibrate the data [17].

In the plasma samples, the 6 dominant lipid classes observed with these two methods are the Lyso-phosphatidylcholines (IS used: C17:0 lyso-phosphatidylcholine), Phosphatidylcholines (IS used: di-C12:0 phosphatidylcholine), Sphingomyelines (IS used: di-C12:0 phosphatidylcholine), Cholesterolesters (IS used: C17:0 cholesterol ester), Triglycerides (IS used: tri-C17:0 glycerol ester), and free fatty acids (IS used: C17:0 FFA). In addition to these lipids, the extracts also contain minor lipids, but these were either not detected (concentration too low relative to very abundant lipids like phosphatidylcholines and triglycerides) or they were not included in data processing. The LC-MS lipid and LC-MS FFA data were processed using the LC-Quan software (Thermo).

#### LC-MS polar

Polar plasma metabolites were analyzed using LC-MS after derivatization (butylation). The metabolites were extracted from 10 µl plasma with 200 µl methanol containing internal standards (deuterated amino acids). After the methanol evaporated, the extract was dissolved in 100 µl n-butanol containing 4 M/l hydrochloric acid and heated to 65°C for 60 min. After freeze drying the extracts were dissolved in 100 µl 0.1% formic acid in water, and 10 µl was injected for LC-MS analysis using a Thermo LTQ equipped with an ESI interface and a Surveyor HPLC system. QC samples, prepared from pooled plasma, were analyzed after every 6^th^ study sample. The mass spectrometer was operated in positive ion mode and data were acquired by scanning from m/z 125 to 1000 at approximately 2 scans/s. The HPLC method consisted of an Intersil ODS 3 column (100×3 mm id) in combination with an acetonitrile gradient (5 to 80% in 20 min at a flow of 0.3 ml/min) in 0.1% formic acid.

Data were processed with TNO comprehensive peak picking software (IMPRESS, [18], [19] to find consistent features in the LC-MS files. These features, after de-isotoping, were used for data processing with Thermo LC-Quan software.

#### GC-MS global

The GC-MS method used for analyzing a broad range of metabolites was identical to the method reported for microbial metabolic profiling [20], except for the sample type. Plasma samples (100 µl) were extracted with methanol and after evaporation the metabolites were derivatized (oximation and silylation). QC samples, prepared from pooled plasma, were analyzed after every 10^th^ study sample.

#### Performance of metabolic profiling platforms

The performance of the applied metabolic profiling platforms is assessed through the frequent analysis of the QC sample [17]. This QC sample, prepared by pooling selected study samples, represents the full biochemical diversity of the study samples and allows the calculation of the analytical precision for all metabolites measured. The QC sample data is also used to correct systematic errors (e.g. batch to batch response differences) by a single point calibration model. Typically, this procedure offers excellent precision for a large majority of metabolites (e.g. 50% of the metabolites have an RSD of less than 10%, 75% with an RSD less than 20%). Metabolites with very high imprecision e.g. RSD>50%, were removed from the data unless large differences between treatment groups were observed. Furthermore, method performance was carefully monitored using multiple internal standards (5 to 10 depending on method, including analogues, 2H and 13C labeled metabolites) and duplicate analysis of samples. The metabolite data used for statistical data analysis in this study met all of our quality requirements.

### Preprocessing of metabolic profiling data

Data for each subject were corrected for the recovery of the IS for injection. Batch to batch differences in data were removed by synchronizing medians of QC-samples per batch. For all platforms, duplicate measurements were combined into a single measurement. When both analytical duplicates had a zero value or when both had a non-zero value, measurements were averaged. The single value was taken when only one of the duplicates was above zero [15]. To avoid trivial results, data were additionally optimized by removing glucose-related metabolites and IS-isotopes in the LC-MS polar data and two glucose metabolites in the GC-MS global data set. The correlation between these glucose peaks and glucose measured by a commercial test kit (Roche Diagnostics GmbH, Mannheim, Germany) on a Hitachi 911 automatic analyzer (Hitachi Instrument Division, Ibaraki-ken, Japan) was 0.97 and 0.98 respectively. Finally, the LC-MS FFA data set contained 14 metabolites, the LC-MS Lipids data set consisted of 61 metabolites, 120 metabolites were included in the LC-MS polar data set and the GC-MS data set contained 137 metabolites.

### Multivariate analysis of metabolic profiling data

#### Two-way analysis: PLS-DA

Partial Least Squares Discriminant analysis (PLS-DA) [21] was used to identify metabolites that differ in their change between day 0 and day 9 in fasted conditions between treatment groups (Figure 1, analysis a). In PLS-DA, a Y-variable containing class membership information is correlated to a data matrix (X-block). The subjects who received the placebo treatment were assigned to class ‘0’ and the subjects who received diclofenac were assigned to class ‘1’. Since the interest was in intra-individual differences between day 0 and day 9, the X-block was defined for each metabolite platform by subtracting the day 0 values from the day 9 values, which removed differences in baseline.

#### Two-way analysis: model validation and optimization

Cross-validation was used to validate the PLS-DA models, using a ‘leave-one-out’ cross-validation scheme [22]. Data of one subject were left out in the first cross-validation step, a PLS-DA model was built, and the treatment class membership of the subject that was left out was predicted. This was repeated until all 19 subjects were left out once. The error rate of the model was determined by comparing the original class membership and the predicted one. The optimal number of LVs was determined based on the minimum value of this error rate. The final fit of the model was made using this number of optimal LVs.

PLS-DA models for which an error rate was found below 35% were optimized by performing metabolite selection based on a jackknife approach [22]. Data of one subject were left out and a PLS-DA model was made using the same number of LVs that was used for the final model. This was repeated until all 19 subjects were left out once. This resulted in 19 sets of regression coefficients, of which the standard deviation was used to determine the relative standard devations (RSDs) of each regression coefficient. Only those metabolites that had a RSD of less than 50% were included in a new data set. This set was used to build a second PLS-DA model. Metabolites that contributed to treatment differences were identified based on absolute regression coefficients of this second model.

#### Three-way analysis: n-PLS-DA

To identify metabolites that differed in changes over time between the treatment groups, it was necessary to discriminate between the time and the metabolite information. Basic multivariate data analysis tools like Principal Component Analysis (PCA; [23], [24], [25], [26]) and Partial Least Squares Discrimant analysis (PLS-DA; [21]) are not sufficient to analyze the data sets, since these methods do not separate the time factor from the metabolites. Therefore, the multi-way generalization of these two-way techniques, nPLS-DA, [27], [28] was used for the analyses. A so called 3-way matrix was created, having size *19*×*J*×*T* where *J* is equal to the number of metabolites of a particular platform and T is equal to the number of time points, which were either the days 0, 2, 4, 7, and 9 (Figure 1, analysis b) or the time points after glucose administration on day 0 and 9 (Figure 1, analysis c). In order to focus the analysis on changes over time within a subject, the day 0 data were subtracted from the day 9 data.

The GC-MS global and LC-MS polar data sets were centered across subjects and followed by scaling within the metabolite-mode *J*, whereas the LC-MS lipids and fatty acids data sets were only centered across subjects. The centering step was performed to remove constants between the subjects, whereas scaling within the metabolite mode resulted in standardized metabolites. By performing the scaling step after the centering step, the prior centering remained unaffected [29], [30], [31].

#### Three-way analysis: model validation and optimization

Cross-validation was used to validate the nPLS-DA models, using a ‘leave-one-subject-out’ cross-validation scheme. Data of one subject (all measurements for all metabolites for one subject) were left out in the first cross-validation step, an nPLS-DA model was built using data of the remaining subjects, and the treatment class membership of the subject that was left out was predicted. This was repeated until all 19 subjects were left out once. The error rate of the model was determined by comparing the original class membership and the predicted one. The optimal number of LVs of the nPLS-DA model was determined based on the minimum value of this error rate.

In order to optimize the nPLS-DA models, metabolite selection has been performed using a jackknife approach. Data of one subject (all measurements for all metabolites for one subject) were left out and an nPLS-DA model was made using the same number of LVs that was used for the final model. This was repeated until all 19 subjects were left out once. This resulted in 19 sets of regression coefficients, of which the standard deviation was used to determine the RSD's of each regression coefficient for each metabolite and each time point. A second nPLS-DA model was build using only those metabolites which showed relatively constant regression coefficients over time.

A permutation test was performed to test whether the treatment differences were indeed true differences similar as described by Bijlsma et al [15]. Therefore, the Y-variable containing class membership information was randomized a 10000 times. For each random vector, a multilevel nPLS-DA model was made using the same (optimal) number of LVs as determined previously. For every nPLS-DA model built, a sum of squares between/sum of squares within ratio (B/W) was calculated for the class assignment predictions. These distributions of random class assignments can be plotted in a histogram and compared to ratio for the original model. The model is classified as ‘bad’ if the B/W of the model is plotted in the lower half of the B/W distribution of random class assignments; the model is classified as ‘moderate’ if the B/W of the model is plotted in the upper half of the B/W distribution of random class assignments; the model is classified as ‘good’ if the B/W of the model is larger than the B/W distribution of random class assignments.

All analyses were performed using Matlab Version 7.0.4 R14 (The Mathworks, Inc.) and the n-way toolbox version 2.11 [32].

### Annotation and Identification of metabolites

The nPLS-DA model resulted in a regression matrix of size J*×*K*, in which J* is the number of metabolites after variable selection. To determine the variables which contributed most to treatment differences, the regression coefficients were sorted by their absolute value in descending order per time point. Since the regression coefficients decreased gradually between the highest and the lowest value due to the use of autoscaled data, there was no sharp cutoff. Therefore, for each time point the first ten peaks with unknown identity were selected and used for metabolite identification.

Metabolites were annotated using an in-house metabolite database containing retention time information, MS spectra (EI for GC-MS data), MS/MS spectra (LC-MS) and accurate mass data (LC-MS) of reference substances. The confidence of identification is 100% unless indicated otherwise. Accurate mass MS and MS/MS data of reference substances and metabolites in the study samples were acquired using Thermo LTQ-FT and Thermo LTQ-Orbitrap instruments.

### Biological interpretation of metabolic profiling data

For each metabolite, the mean treatment effect difference between day 9 and day 0 during the OGTT time course was calculated as follows:
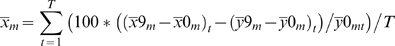
Where

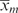
 = mean treatment effect for metabolite *m* in differences between day 9 and day 0 (%)

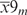
 = mean intensity for metabolite *m* on day 9 for the diclofenac treatment group

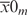
 = mean intensity for metabolite *m* on day 0 for the diclofenac treatment group

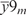
 = mean intensity for metabolite *m* on day 9 for the placebo group

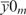
 = mean intensity for metabolite *m* on day 0 for the placebo group
- T = total number of time points (t = 1 … 8)

Ranges in terms of percentages were calculated per metabolite as the minimum and maximum value of the treatment effects calculated per time point. The minimum and maximum value of treatment effects were calculated per time point per metabolite and these values were used to determine the ranges of treatment effects in terms of percentages.

Detailed pathway and biological network analysis was performed in Metacore version 4.3 (GeneGo Inc., St. Joseph, MI, USA). Only curated interactions were used for biological network analysis. The following metabolites were not available in Metacore and therefore not used for pathway and network analysis: 1,2-diglyceride (C36:2), 2,3,4-trihydroxybutanoic acid and 2-amino-2-methyl butanoic acid, 1-aminocyclopentanecarboxylic acid. Pathway maps were edited in Mapeditor (GeneGo Inc., St. Joseph, MI, USA) version 2.1.0.

## Results

### Statistical selection of relevant metabolites for diclofenac treatment

Metabolites that changed due to diclofenac treatment were identified using various multivariate comparisons. Table 3 shows the results of these multivariate analyses of the different datasets derived from the four metabolic profiling platforms.

**Table 3:** Multivariate data analysis of various metabolic profiling datasets.

Method	GC-MS	LC-MS	LC-MS	LC-MS
	global	polar	Lipids	FFA
# metabolites	137	130	61	14
PLS-DA				
*Day 9 vs Day 0*	42.00%	53.00%	37.00%	37.00%
n-PLS-DA				
*Day 0, 2, 4, 7 and 9*	57.00%	42.00%	52.50%	47.00%
n-PLS-DA				
*Day 9 vs Day 0; 0–15–30–45–60–90–120 and 180 min*	31.50%	31.50%	21.00%	31.50%
n-PLS-DA after metabolite selection				
*Day 9 vs Day 0; 0–15–30–45–60–90–120 and 180 min*	# = 77	# = 31	# = 25	NA
	10.50%	5.00%	16.00%	

The results of the different multivariate models are expressed as error rates.

Metabolite selection was only applied if the error rate of the original model of the complete dataset was below 35% and the dataset contained more than 50 metabolites. NA, not analyzed.

A comparison of the fasting state metabolomes between the subjects treated with placebo and diclofenac on day 9 compared to day 0 resulted in PLS-DA models (Figure 1, analysis a) with high error rates. This indicated that there was no significant difference in fasted plasma samples.

Expanding the n-PLS-DA models with the metabolic profiling data from the plasma samples taken in fasted conditions on several intermediate days (Figure 1, analysis b) during the intervention for the various metabolic profiling platforms also resulted in high error rates. This confirms that differences in metabolic changes could not be detected between subjects treated with placebo and diclofenac in the fasted (homeostatic) condition.

Metabolic perturbation by the OGTT improved the metabolic profiling-based differentiation between the treatments. The n-PLS-DA models on plasma samples taken at 8 point time course after the glucose administration to subjects on day 9 vs day 0 (Figure 1, analysis c) resulted in improved error rates. This was true for all metabolic profiling platforms as compared to PLS-DA and n-PLS-DA models based on plasma in fasted conditions, between the control and anti-inflammatory treatment. The n-PLS-DA models for metabolome data from GC-MS global, LC-MS polar and LC-MS lipids were analyzed further with metabolite selection methods. The n-PLS-DA models after metabolite selection for GC-MS global and LC-MS polar metabolome data resulted in models with error rates of 10.5% and 5.0% respectively, indicating that the GC-MS global model and the LC-MS polar model only misclassified respectively 2 and 1 persons of the total 19. The model of the LC-MS lipids-dataset resulted in a model with an error rate of 16%, indicating that 3 out of 19 total subjects were misclassified. Permutation tests were performed on these 3 models to validate the significance of the treatment differences. The results of the GC-MS global and LC-MS polar permutation test were ‘good’, in contrast to the results for the LC-MS lipids model which were ‘moderate’.

Overall, significant metabolic changes due to the treatment could be detected only in the metabolic profiling data of the OGTT time course. Only metabolome data from the models with ‘good’ results for the permutation test, thus the LC-MS polar and GC-MS global models (after metabolite selection), were used for further interpretation in the statistical analysis.

### Metabolite identification

The metabolites with the highest absolute regression coefficients per time point (thus 0, 15, 30, 45, 60, 90, 120 and 180 minutes) were selected from the LC-MS polar and GC-MS global n-PLS-DA models as being most discriminative between subjects treated with diclofenac and placebo. The intersection of the regression coefficients per time point resulted in a total of 15 unique metabolites from the GC-MS global dataset and in a total of 24 unique metabolites from the LC-MS polar dataset for metabolite identification. Ultimately, 69% of the selected metabolites could be identified (14 out of 15 in the GC-MS global dataset; 13 out of 24 in the LC-MS polar dataset). Table 4 lists the most discriminating metabolites that could be identified. Only the identified metabolites were used for further interpretation.

**Table 4:** Overview of most discriminating metabolites, their treatment effect and their metabolite response in the OGTT time course.

	Metabolite	Treatment effect (%): Diclofenac vs placebo			
		Mean	Range		Response type
GC-MS	Uric acid	−12	−18	1	A
	1,2-diglyceride (C36:2)	−18	−28	−9	A
	Proline	9	6	13	A
	Isoleucine	−18	−22	−13	A
	1-aminocyclopentanecarboxylic acid	−2	−5	2	A
	Threonine	−13	−15	−11	A
	4-Hydroxyproline	−59	−72	−51	A
	2,3,4-Trihydroxybutanoic acid	15	9	24	A
	Aminoadipic acid	−26	−43	−16	A
	Arabitol, ribitol, or xylitol	10	5	13	A
	Ornithine	−12	−20	2	A
	Mannose or galactose	10	6	14	A
	Palmitoleic acid (C16:1)	23	−2	55	A
	Palmitic acid (C16:0)	6	−13	24	A
LC-MS	Isoleucine	−23	−34	−15	A
	Glycine	8			B
	2-Amino-2-methyl butanoic acid	−4	−9	−1	A
	5-Oxoproline	14			B
	1-Aminocyclopentanecarboxylic acid	−69	−91	−35	A
	4-Hydroxyproline	−52	−61	−38	A
	Isoleucine & Leucine (not resolved)	−8	−13	−1	A
	Hippuric acid	63	48	75	A
	5-Oxoproline (acetonitrile adduct)	8			B
	Aspartic acid	9			B
	Glutamic acid	4			B
	Citric acid	−10	−18	6	A

Per analytical platform the metabolites are ranked according to their importance to the model, thus uric acid and isoleucine contributed most in the discrimination between the treatment groups.

**Figure 2:**
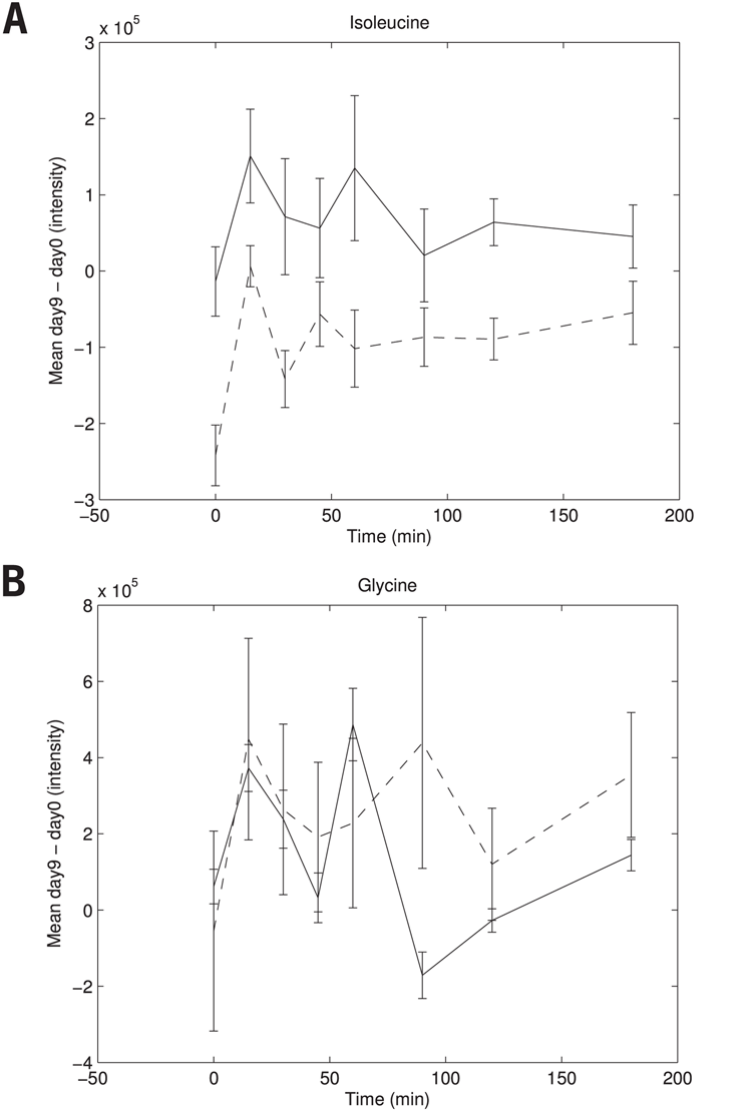
OGTT time course mean metabolite response with standard error on day 9 corrected for concentrations on day 0 for subjects on placebo and diclofenac treatment. A) for the metabolite isoleucine that contributes to treatment differences over the whole time course and B) for the metabolite glycine that contributes to treatment differences only in the second part of the time course. *Legend to* *Figure 2*: Dashed line: Diclofenac treated subjects; Solid line: Placebo treated subjects.

Only identified metabolites are shown in Table 4. The column ‘response type’ refers to Figure 2; metabolites with response type A showed a treatment difference over the whole time course and metabolites with response type B showed a treatment difference only in the second part of the time course (a response of the metabolite in the placebo group, whereas no change in the diclofenac treated group). For metabolites with response type B the mean is calculated over the time points with a treatment difference.

### Analysis of metabolite response during OGTT time course

The metabolite response was tracked by plotting the mean difference between day 9 and day 0 per time point per treatment group. In general, two different metabolite challenge test responses were distinguished as illustrated in Figure 2. Most of the selected metabolites (81%, Table 4) showed a difference in offset that is constant during the OGTT time course (Figure 2A, response type A). In other words, these metabolites are discriminating between the treated and untreated subjects independent of time during the OGTT time course. This indicates that only minor differences exist between the treatment groups and that these differences can only be identified by repeated measurements. Indeed, time independent PLS-DA analysis yielded a similar error rate (11%).

Some of the selected metabolites (19%), however, showed only a contribution to treatment differences in the second part of the OGTT time course (Figure 2B, response type B). This indicates that these 4 metabolites only differed between treatments when challenging the metabolic situation, leading to alterations in *dynamic* response to the perturbation. Indeed, time independent PLS-DA analysis increased the error rate of the LC-MS polar model (26%).

Furthermore, Table 4 shows the semi-quantified treatment effects, expressed as the mean change (in %) over time. The majority of the metabolites that had a constant contribution to treatment differences over time (categorized as response type A) showed a decreased concentration in plasma in response to diclofenac treatment compared to subjects treated with placebo. Several amino acids (n = 6), organic acids (n = 7), carbohydrates (n = 2) and fatty acids & lipids (n = 3) were categorized with a response type A. Some metabolites were identified as being discriminating between the treatments in data from both analytical platforms (isoleucine, 1-aminocyclopentanecarboxylic acid and 4-hydroxyproline), validating their contribution to the differences between the treatment groups.

All metabolites that specifically showed a dynamic response to the perturbation (categorized as response type B), showed higher concentrations in the diclofenac treated group. Based on plots of mean changes over time, it appeared that mean concentrations of diclofenac treated subjects remained constant, whereas mean concentrations of placebo treated subjects dropped during a specific phase of the time course (Figure 2b). The amino acids glycine, aspartic acid and glutamic acid and the organic acid 5-oxoproline were categorized with a response type B.

## Discussion

A primary goal of research into lifestyle associated diseases is to optimize health so that the onset of disease can be prevented or delayed. In identifying the key changes involved in the development of lifestyle associated diseases, experimental approaches have to deal with large inter-individual variety and the robustness of homeostasis. The current study deliberately recruited healthy overweight men with slightly increased inflammation parameters and successfully applied a relatively mild anti-inflammatory intervention so that only subtle changes were to be expected. The study authors aimed to demonstrate that an experimental design using metabolic profiling in concert with a challenge test is a good strategy for the unraveling of biomarkers in intervention studies where only subtle changes are to be expected.

The conventional metabolic profiling approach of measuring blood samples in fasting conditions – even in a time course – and the classical biomarkers (i.e. glucose, insulin, sialic acid, HOMA index, and adiponectin; van Erk et al, in prep) were not able to reveal changes in response to diclofenac treatment. In this study, the subtle metabolic changes resulting from diclofenac treatment could only be determined using an OGTT time course. This can have at least two reasons. Firstly, most of the treatment differences became significant by repeated confirmation of subtle homeostatic alterations in metabolite concentrations (metabolites with response type A) without dealing with any day-to-day variations like in the ‘long-term’ fasted time course. Secondly, by perturbing a homeostatic metabolic situation, metabolite differences with a dynamic response to the oral glucose tolerance test became visible (response type B metabolites).

Diclofenac is known to inhibit and activate several enzymes and transporters [33]–[41]. CD13 is a broad specificity aminopeptidase that cleaves specifically the N-terminal bound neutral amino acids from oligopeptides. The inhibition of the enzyme aminopeptidase N (CD13) by diclofenac corresponds to the lower plasma concentrations of several neutral amino acids such as L-isoleucine, L-threonine, and L-leucine. Such consistent lowering is reflected in a type A response.

In the current study, the diclofenac intervention applied was successfully shown by significantly reduced concentrations of PGE2 (see Materials and Methods). Metabolic profiling revealed that the diclofenac treatment resulted in lower plasma levels of uric acid. Elevated serum uric acid levels are positively associated with metabolic syndrome, insulin resistance and diabetes type II [42], [43] and has been proposed as risk factor for hypertension and cardiovascular diseases [44], [45]. Subsequently, elevated levels of uric acid are associated with inflammation and oxidative stress [42], [43], [46]. The current results suggest that inhibition of cyclooxygenase mediated inflammation (shown by significantly reduced concentrations of PGE2) could be associated with reduced concentrations of uric acid and therefore might lead to a reduction of risk on several metabolic diseases. However, this needs to be further explored.

**Figure 3:**
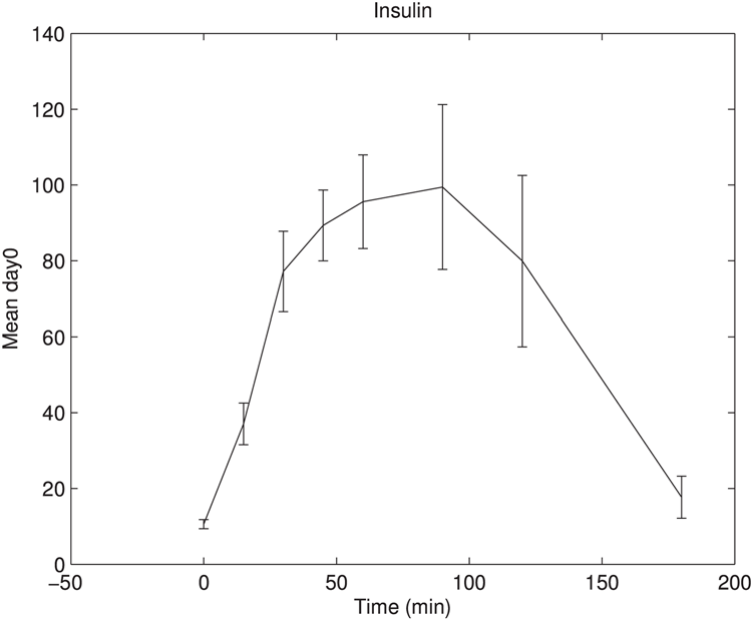
Day 0 insulin response in OGTT time course. Day 0 insulin mean response with standard error is shown in OGTT time course for all subjects. Insulin concentrations show a maximum at ∼65 minutes after glucose intake.

Most interestingly in this study are the metabolites that showed a differential response between the treatments groups to the OGTT (metabolites identified with a response type B). All metabolites with response type B showed the largest difference between the treatments at time point 90 and/or 120 minutes after intake of glucose. Insulin peaks at an average of 65 minutes after glucose intake (Figure 3). This suggests that differences in response to the OGTT may be attributed to the action of insulin.

**Figure 4:**
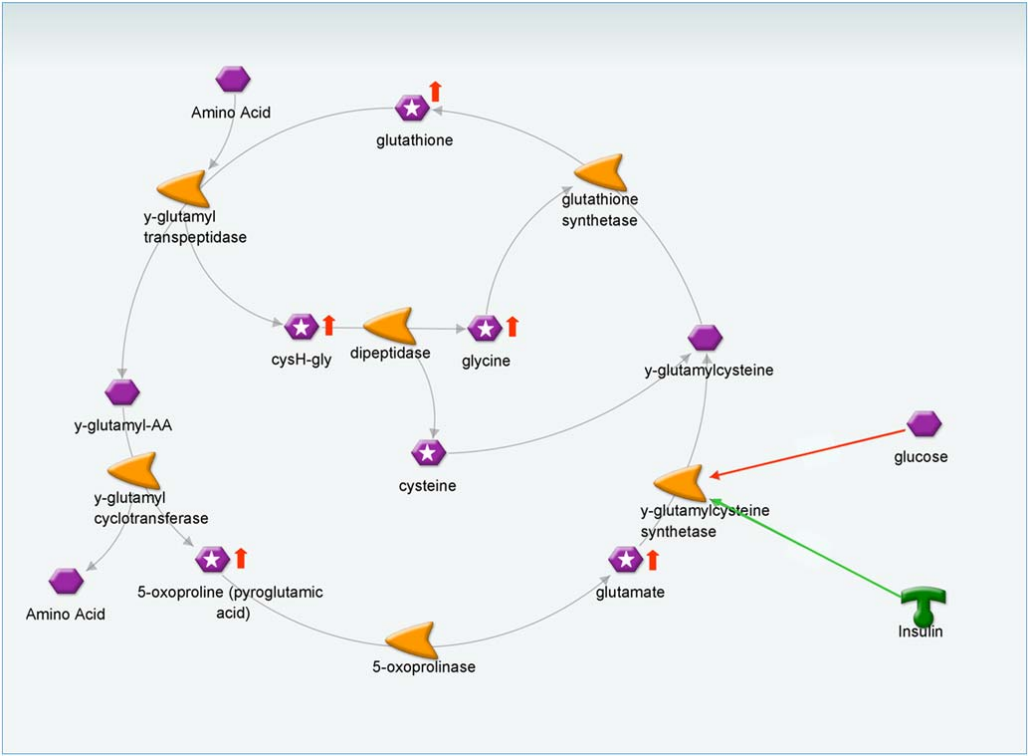
Glutathione synthesis pathway and its connection to glucose and insulin. High levels of glucose inhibit and high levels of insulin activate glutathione synthesis via the enzyme γ-glutamylcysteine synthetase. *Legend to* *Figure 4*: Connection arrows with color red represent inhibition and color green represent activation. Purple hexagons represent metabolites; purple hexagons with white star represent metabolites measured with one of the metabolic profiling platforms; orange symbols represent enzymes. Red arrows upwards indicate that higher plasma concentration levels were found in the diclofenac treated group in response to oral glucose tolerance test. Abbreviations: AA, amino acid; Cys-Gly, cysteinylglycine. These figures were created by using MapEditor version 2.1.0 (GeneGo Inc, St Joseph, MI).

**Figure 5:**
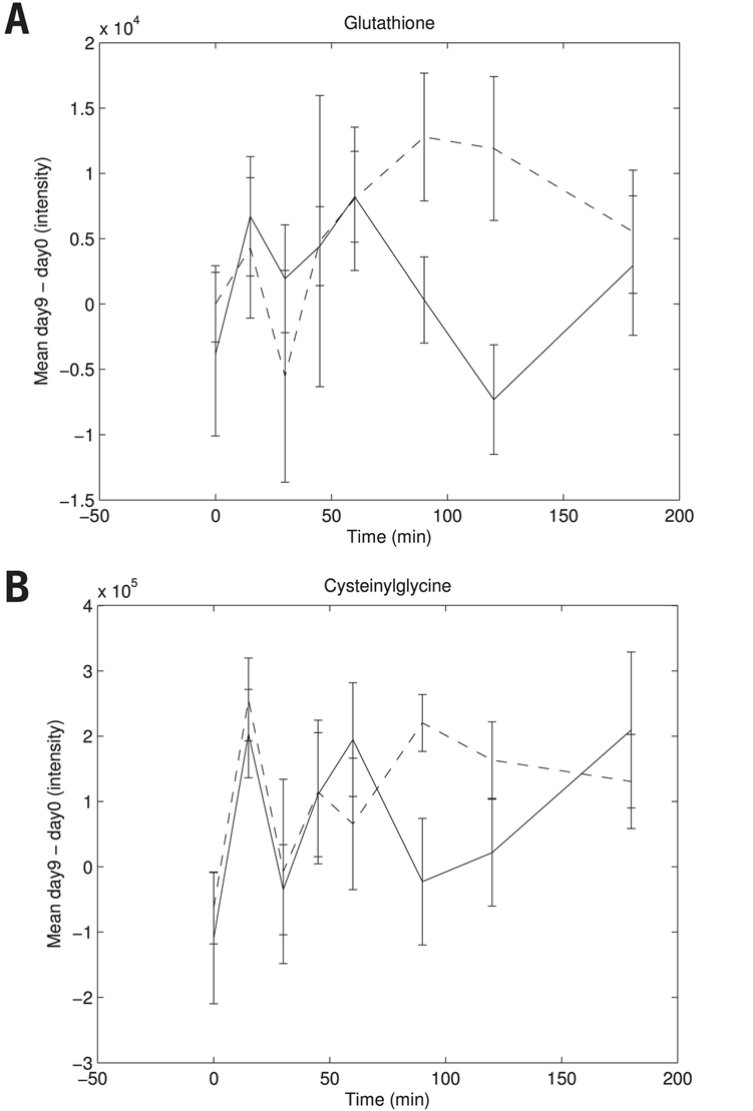
Dynamic response of glutathione synthesis pathway intermediates in OGTT time course. A) glutathione mean response with standard error on day 9 corrected for concentrations on day 0 for subjects on placebo and diclofenac treatment. Glutathione showed a treatment difference only in the second part of the time course. B) Cysteinylglycine mean response with standard error on day 9 corrected for concentrations on day 0 for subjects on placebo and diclofenac treatment. Cysteinylglycine showed a treatment difference only in the second part of the time course. *Legend to* *Figure 5*: Dashed line: Diclofenac treated subjects; Solid line: Placebo treated subjects.

**Figure 6:**
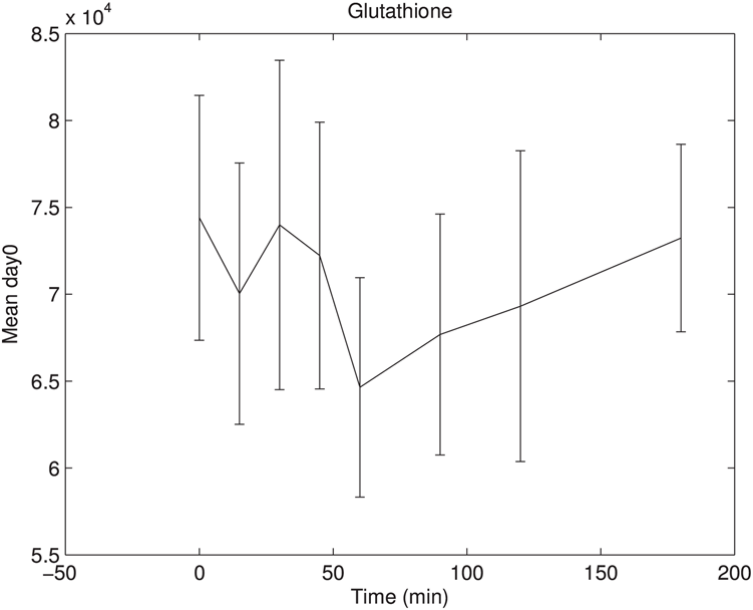
Day 0 glutathione response in OGTT time course. Day 0 glutathione mean response with standard error is shown in OGTT time course for all subjects. Glutathione showed declined concentrations in the first part of the time course with a minimum concentration at 60 minutes after glucose intake. In the second part of the time course concentrations increase again.

Of the 26 peaks that were most discriminative in the nPLS-DA models (Table 3), five peaks were found with this response type B profile of which two were annotated as 5-oxoproline and the others as the amino acids glycine, aspartic acid and glutamic acid. Three of these - 5-oxoproline, glycine and glutamic acid - are known to be involved in the glutathione synthesis pathway (Figure 4). Therefore, the response of other intermediates in the glutathione synthesis pathway was also studied. It appeared that glutathione and cysteinylglycine showed a similar dynamic response to the OGTT as the other type B responders (Figure 5), with the exception of plasma cysteine. Figure 4 provides and overview of the glutathione synthesis pathway and its relationship to glucose and insulin. Higher clearance of plasma L-5-oxoproline is known in case of lower GSH synthesis [47]. GSH synthesis is predominantly regulated by activity of γ-glutamylcysteine synthetase (GCS) and availability of the rate-limiting substrate cysteine [48]. Interestingly, it is known that insulin action increases and glucose decreases the regulation of GSH synthesis by GCS [48]–[50]. In this study, the average plasma glutathione concentrations declined to their minimum concentration at 60 minutes after glucose intake and increased again at 90 to 120 minutes after glucose intake (Figure 6). In the current study, the control group showed significant lower concentrations of glutathione synthesis pathway intermediates at 90 to 120 minutes after glucose intake compared to diclofenac treated subjects. This might indicate that diclofenac treatment resulted in a higher GSH synthesis response after the glucose bolus, which might be related to altered insulin signaling with diclofenac treatment. It has been shown earlier that a selective inhibition of cyclooxygenase-2 results in increased insulin sensitivity in overweight or obese subjects [51]. However, no differences were found in classical insulin sensitivity indexes (HOMA index, ISIcomp, MCRest and Gutt-index) between diclofenac and placebo treated subjects in this study. A possible explanation is that the combination of multiple metabolites as biomarker in concert with an oral glucose tolerance test allows for an earlier detection of changes in insulin sensitivity, however this is speculation at this stage and should be further explored.

This first exploratory study shows that subtle metabolic changes resulting from an anti-inflammatory treatment could only be determined using a metabolic perturbation test in a well-designed clinical study using metabolic profiling analysis. Differences in dynamic response to the challenge (response type B metabolites) might be derived from insulin regulated processes such as the insulin regulated glutathione synthesis pathway. Our study demonstrates that the use of metabolic profiling in concert with a challenge test may open new avenues for biomarker discovery that could be useful in developing preventive strategies for lifestyle associated diseases.

## Supporting Information

Table S1 GCMS global platform nr 1 LCMS polar platform nr 2 LCMS lipids platform nr 3 LCMS Free Fatty Acids platform nr 4 Abbreviations: LPC, lysophosphatidylcholine; PC, phosphatidylcholine; SPM, sphingomyeline; ChE, cholesterolester; TG, triglyceride; FA, fatty acid.
(0.52 MB DOC)
