# Supplementary material for: Metabolic Profiling of the Response to an Oral Glucose Tolerance Test Detects Subtle Metabolic Changes
Source: PLoS One. 2009 Feb 26;4(2):e4525. doi: 10.1371/journal.pone.0004525 (PMC2643463; doi:10.1371/journal.pone.0004525)
Supplement: Table S1 — GCMS global platform nr 1 LCMS polar platform nr 2 LCMS lipids platform nr 3 LCMS Free Fatty Acids platform nr 4 Abbreviations: LPC, lysophosphatidylcholine; PC, phosphatidylcholine; SPM, sphingomyeline; ChE, cholesterolester; TG, triglyceride; FA, fatty acid. (0.52 MB DOC) [file pone.0004525.s001.doc]

**Table S1**

Overview of metabolites analyzed with the 4 different metabolic profiling platforms

GCMS global platform nr 1

LCMS polar platform nr 2

LCMS lipids platform nr 3

LCMS Free Fatty Acids platform nr 4

Abbreviations: LPC, lysophosphatidylcholine; PC, phosphatidylcholine; SPM, sphingomyeline; ChE, cholesterolester; TG, triglyceride; FA, fatty acid.

| **Metabolite #** | **Metabolite ID** | **HMDB ID** | **Platform nr** |
| --- | --- | --- | --- |
| 1 | Lactic acid | [HMDB00190](http://hmdb.ca/scripts/show_card.cgi?METABOCARD=HMDB00190.txt) | 1 |
| 2 | Alanine | [HMDB00161](http://hmdb.ca/scripts/show_card.cgi?METABOCARD=HMDB00161.txt) | 1 |
| 3 | Pyruvic acid | [HMDB00243](http://hmdb.ca/scripts/show_card.cgi?METABOCARD=HMDB00243.txt) | 1 |
| 4 | Benzoylalcohol | [HMDB03119](http://hmdb.ca/scripts/show_card.cgi?METABOCARD=HMDB03119.txt) | 1 |
| 5 | unknown 1 |  | 1 |
| 6 | 3-Hydroxybutanoic acid | [HMDB00357](http://www.hmdb.ca/scripts/show_card.cgi?METABOCARD=HMDB00357) | 1 |
| 7 | Phosphoric acid monomethylester | [HMDB02142](http://hmdb.ca/scripts/show_card.cgi?METABOCARD=HMDB02142.txt) | 1 |
| 8 | unknown 2 |  | 1 |
| 9 | Valine | [HMDB00883](http://hmdb.ca/scripts/show_card.cgi?METABOCARD=HMDB00883.txt) | 1 |
| 10 | 4-methyl-2-oxovaleric acid |  | 1 |
| 11 | Leucine | [HMDB00687](http://hmdb.ca/scripts/show_card.cgi?METABOCARD=HMDB00687.txt) | 1 |
| 12 | Glycerol | [HMDB00131](http://hmdb.ca/scripts/show_card.cgi?METABOCARD=HMDB00131.txt) | 1 |
| 13 | Phosphate | [HMDB01429](http://hmdb.ca/scripts/show_card.cgi?METABOCARD=HMDB01429.txt) | 1 |
| 14 | Proline | [HMDB00162](http://hmdb.ca/scripts/show_card.cgi?METABOCARD=HMDB00162.txt) | 1 |
| 15 | Isoleucine | [HMDB00172](http://hmdb.ca/scripts/show_card.cgi?METABOCARD=HMDB00172.txt) | 1 |
| 16 | Glycine | [HMDB00123](http://hmdb.ca/scripts/show_card.cgi?METABOCARD=HMDB00123.txt) | 1 |
| 17 | Succinic acid | [HMDB00254](http://hmdb.ca/scripts/show_card.cgi?METABOCARD=HMDB00254.txt) | 1 |
| 18 | 1-aminocyclopentanecarboxylic acid |  | 1 |
| 19 | Glyceric acid | [HMDB00139](http://hmdb.ca/scripts/show_card.cgi?METABOCARD=HMDB00139.txt) | 1 |
| 20 | unknown 3 |  | 1 |
| 21 | unknown 4 |  | 1 |
| 22 | Pipecolinic acid | [HMDB00070](http://hmdb.ca/scripts/show_card.cgi?METABOCARD=HMDB00070.txt) | 1 |
| 23 | Serine | [HMDB00187](http://hmdb.ca/scripts/show_card.cgi?METABOCARD=HMDB00187.txt) | 1 |
| 24 | unknown 5 |  | 1 |
| 25 | Threonine | [HMDB00167](http://hmdb.ca/scripts/show_card.cgi?METABOCARD=HMDB00167.txt) | 1 |
| 26 | unknown 6 |  | 1 |
| 27 | by-product of alanine | [HMDB00161](http://hmdb.ca/scripts/show_card.cgi?METABOCARD=HMDB00161.txt) | 1 |
| 28 | unknown 7 |  | 1 |
| 29 | unknown 8 |  | 1 |
| 30 | Beta-Alanine | [HMDB00056](http://hmdb.ca/scripts/show_card.cgi?METABOCARD=HMDB00056.txt) | 1 |
| 31 | unknown 9 |  | 1 |
| 32 | unknown 10 |  | 1 |
| 33 | unknown 11 |  | 1 |
| 34 | Aminomalonic acid | [HMDB01147](http://www.hmdb.ca/scripts/show_card.cgi?METABOCARD=HMDB01147) | 1 |
| 35 | unknown 12 |  | 1 |
| 36 | Malic acid | [HMDB00156](http://hmdb.ca/scripts/show_card.cgi?METABOCARD=HMDB00156.txt) | 1 |
| 37 | N-acetylaminomalonic acid |  | 1 |
| 38 | Methionine | [HMDB00696](http://hmdb.ca/scripts/show_card.cgi?METABOCARD=HMDB00696.txt) | 1 |
| 39 | Glutamine degr. internal amide | [HMDB02142](http://hmdb.ca/scripts/show_card.cgi?METABOCARD=HMDB02142.txt) | 1 |
| 40 | Meso-erythrytol |  | 1 |
| 41 | Aspartic acid | [HMDB00191](http://hmdb.ca/scripts/show_card.cgi?METABOCARD=HMDB00191.txt) | 1 |
| 42 | 4-Hydroxyproline | [HMDB00725](http://www.hmdb.ca/scripts/show_card.cgi?METABOCARD=HMDB00725) | 1 |
| 43 | Creatinine | [HMDB00562](http://hmdb.ca/scripts/show_card.cgi?METABOCARD=HMDB00562.txt) | 1 |
| 44 | Cysteine | [HMDB00574](http://hmdb.ca/scripts/show_card.cgi?METABOCARD=HMDB00574.txt) | 1 |
| 45 | Erythronic acid | [HMDB00613](http://hmdb.ca/scripts/show_card.cgi?METABOCARD=HMDB00613.txt) | 1 |
| 46 | 2,3,4-trihydroxybutanoic acid | [HMDB00613](http://www.hmdb.ca/scripts/show_card.cgi?METABOCARD=HMDB00613) | 1 |
| 47 | unknown 13 |  | 1 |
| 48 | unknown 14 |  | 1 |
| 49 | Phenylalanine | [HMDB00159](http://hmdb.ca/scripts/show_card.cgi?METABOCARD=HMDB00159.txt) | 1 |
| 50 | Glutamic acid | [HMDB03339](http://hmdb.ca/scripts/show_card.cgi?METABOCARD=HMDB03339.txt) | 1 |
| 51 | alpha-Ketoglutaric acid | [HMDB00208](http://www.hmdb.ca/scripts/show_card.cgi?METABOCARD=HMDB00208) | 1 |
| 52 | unknown 15 |  | 1 |
| 53 | unknown 16 |  | 1 |
| 54 | Asparagine | [HMDB00168](http://hmdb.ca/scripts/show_card.cgi?METABOCARD=HMDB00168.txt) | 1 |
| 55 | unknown 17 |  | 1 |
| 56 | unknown 18 |  | 1 |
| 57 | unknown 19 |  | 1 |
| 58 | unknown 20 |  | 1 |
| 59 | alpha-Aminoadipic acid | [HMDB00510](http://www.hmdb.ca/scripts/show_card.cgi?METABOCARD=HMDB00510) | 1 |
| 60 | unknown 21 |  | 1 |
| 61 | Xylose | [HMDB00098](http://hmdb.ca/scripts/show_card.cgi?METABOCARD=HMDB00098.txt) | 1 |
| 62 | Arabinose | [HMDB00646](http://www.hmdb.ca/scripts/show_card.cgi?METABOCARD=HMDB00646) | 1 |
| 63 | Arabitol, Ribitol, or Xylitol | [HMDB01851 or HMDB00508 or HMDB02917](http://www.hmdb.ca/scripts/show_card.cgi?METABOCARD=HMDB01851) | 1 |
| 64 | Ribose | [HMDB00283](http://hmdb.ca/scripts/show_card.cgi?METABOCARD=HMDB00283.txt) | 1 |
| 65 | unknown 21 |  | 1 |
| 66 | unknown 22 |  | 1 |
| 67 | sn-Glycerol-3-Phosphate | [HMDB02722](http://hmdb.ca/scripts/show_card.cgi?METABOCARD=HMDB02722.txt) | 1 |
| 68 | Glutamine | [HMDB02142](http://hmdb.ca/scripts/show_card.cgi?METABOCARD=HMDB02142.txt) | 1 |
| 69 | HypoXanthine | [HMDB00157](http://hmdb.ca/scripts/show_card.cgi?METABOCARD=HMDB00157.txt) | 1 |
| 70 | 3-Methylhistidine | [HMDB00479](http://hmdb.ca/scripts/show_card.cgi?METABOCARD=HMDB00479.txt) | 1 |
| 71 | Ornithine | [HMDB00214](http://hmdb.ca/scripts/show_card.cgi?METABOCARD=HMDB00214.txt) | 1 |
| 72 | Citric acid | [HMDB00094](http://hmdb.ca/scripts/show_card.cgi?METABOCARD=HMDB00094.txt) | 1 |
| 73 | 1-Deoxyglucose |  | 1 |
| 74 | unknown 23 |  | 1 |
| 75 | Histidine | [HMDB00177](http://hmdb.ca/scripts/show_card.cgi?METABOCARD=HMDB00177.txt) | 1 |
| 76 | Lysine | [HMDB00182](http://hmdb.ca/scripts/show_card.cgi?METABOCARD=HMDB00182.txt) | 1 |
| 77 | Tyrosine | [HMDB00158](http://hmdb.ca/scripts/show_card.cgi?METABOCARD=HMDB00158.txt) | 1 |
| 78 | Fructose | [HMDB00660](http://hmdb.ca/scripts/show_card.cgi?METABOCARD=HMDB00660.txt) | 1 |
| 79 | Mannose | [HMDB00169](http://hmdb.ca/scripts/show_card.cgi?METABOCARD=HMDB00169.txt) | 1 |
| 80 | Glucose peak 1 | [HMDB00122](http://hmdb.ca/scripts/show_card.cgi?METABOCARD=HMDB00122.txt) | 1 |
| 81 | C6-aldose (mannose or galactose) | [HMDB00169 or HMDB00143](http://hmdb.ca/scripts/show_card.cgi?METABOCARD=HMDB00169.txt) | 1 |
| 82 | unknown 24 |  | 1 |
| 83 | Glucose peak 2 | [HMDB00122](http://hmdb.ca/scripts/show_card.cgi?METABOCARD=HMDB00122.txt) | 1 |
| 84 | unknown 25 |  | 1 |
| 85 | C16:1 fatty acid | [HMDB03229](http://www.hmdb.ca/scripts/show_card.cgi?METABOCARD=HMDB03229) | 1 |
| 86 | C16:0 fatty acid | [HMDB00220](http://www.hmdb.ca/scripts/show_card.cgi?METABOCARD=HMDB00220) | 1 |
| 87 | unknown 26 |  | 1 |
| 88 | unknown 27 |  | 1 |
| 89 | Inositol |  | 1 |
| 90 | unknown 28 |  | 1 |
| 91 | unknown 29 |  | 1 |
| 92 | Myo-inositol | [HMDB00211](http://hmdb.ca/scripts/show_card.cgi?METABOCARD=HMDB00211.txt) | 1 |
| 93 | Uric acid | [HMDB00289](http://hmdb.ca/scripts/show_card.cgi?METABOCARD=HMDB00289.txt) | 1 |
| 94 | C18:2 fatty acid | [HMDB00673](http://www.hmdb.ca/scripts/show_card.cgi?METABOCARD=HMDB00673) | 1 |
| 95 | C18:1 fatty acid peak 1 | [HMDB00207](http://www.hmdb.ca/scripts/show_card.cgi?METABOCARD=HMDB00207) | 1 |
| 96 | C18:1 fatty acid peak2 | [HMDB00208](http://www.hmdb.ca/scripts/show_card.cgi?METABOCARD=HMDB00207) | 1 |
| 97 | Tryptophan | [HMDB00929](http://hmdb.ca/scripts/show_card.cgi?METABOCARD=HMDB00929.txt) | 1 |
| 98 | C18:0 fatty acid | [HMDB00827](http://www.hmdb.ca/scripts/show_card.cgi?METABOCARD=HMDB00827) | 1 |
| 99 | unknown 30 |  | 1 |
| 100 | unknown 31 |  | 1 |
| 101 | unknown 32 |  | 1 |
| 102 | Pseudo uridine | [HMDB00767](http://hmdb.ca/scripts/show_card.cgi?METABOCARD=HMDB00767.txt) | 1 |
| 103 | unknown 33 |  | 1 |
| 104 | unknown 34 |  | 1 |
| 105 | unknown 35 |  | 1 |
| 106 | unknown 36 |  | 1 |
| 107 | C16:0 Glycerol ester |  | 1 |
| 108 | C18:2 Glycerol ester |  | 1 |
| 109 | C18:1 Glycerol ester |  | 1 |
| 110 | C18:0 Glycerol ester |  | 1 |
| 111 | Maltose | [HMDB00163](http://www.hmdb.ca/scripts/show_card.cgi?METABOCARD=HMDB00163) | 1 |
| 112 | unknown 37 |  | 1 |
| 113 | unknown 38 |  | 1 |
| 114 | Vitamin E | [HMDB01893](http://www.hmdb.ca/scripts/show_card.cgi?METABOCARD=HMDB01893) | 1 |
| 115 | Cholesterol | [HMDB00067](http://hmdb.ca/scripts/show_card.cgi?METABOCARD=HMDB00067.txt) | 1 |
| 116 | Dihydrocholesterol | [HMDB00908](http://www.hmdb.ca/scripts/show_card.cgi?METABOCARD=HMDB00908) | 1 |
| 117 | unknown 39 |  | 1 |
| 118 | unknown 40 |  | 1 |
| 119 | unknown 41 |  | 1 |
| 120 | unknown 42 |  | 1 |
| 121 | unknown 43 |  | 1 |
| 122 | unknown 44 |  | 1 |
| 123 | unknown 45 |  | 1 |
| 124 | unknown 46 |  | 1 |
| 125 | unknown 47 |  | 1 |
| 126 | C18:0 Sphingomyeline |  | 1 |
| 127 | unknown 48 |  | 1 |
| 128 | unknown 49 |  | 1 |
| 129 | unknown 50 |  | 1 |
| 130 | unknown 51 |  | 1 |
| 131 | unknown 52 |  | 1 |
| 132 | unknown 53 |  | 1 |
| 133 | unknown 54 |  | 1 |
| 134 | unknown 55 |  | 1 |
| 135 | 1,2-diglyceride (C36:2) |  |  |
| 136 | unknown 56 |  | 1 |
| 137 | 1,3-Diglyceride |  | 1 |
| 138 | unknown 57 |  | 1 |
| 139 | unknown 58 |  | 1 |
| 140 | Isoleucine + Leucine (not resolved) | [HMDB00172 or HMDB00687](http://hmdb.ca/scripts/show_card.cgi?METABOCARD=HMDB00687.txt) | 2 |
| 141 | Glycine | [HMDB00123](http://hmdb.ca/scripts/show_card.cgi?METABOCARD=HMDB00123.txt) | 2 |
| 142 | Alanine | [HMDB00161](http://hmdb.ca/scripts/show_card.cgi?METABOCARD=HMDB00161.txt) | 2 |
| 143 | 4-Aminobutanoic acid | [HMDB00112](http://www.hmdb.ca/scripts/show_card.cgi?METABOCARD=HMDB00112) | 2 |
| 144 | Serine | [HMDB00187](http://www.hmdb.ca/scripts/show_card.cgi?METABOCARD=HMDB00187) | 2 |
| 145 | unknown 59 |  | 2 |
| 146 | Proline | [HMDB00162](http://hmdb.ca/scripts/show_card.cgi?METABOCARD=HMDB00162.txt) | 2 |
| 147 | 2-amino-2-methyl butanoic acid | [HMDB01906](http://hmdb.ca/scripts/show_card.cgi?METABOCARD=HMDB01906.txt) | 2 |
| 148 | Valine | [HMDB00883](http://hmdb.ca/scripts/show_card.cgi?METABOCARD=HMDB00883.txt) | 2 |
| 149 | Threonine | [HMDB00167](http://hmdb.ca/scripts/show_card.cgi?METABOCARD=HMDB00167.txt) | 2 |
| 150 | Cysteine | [HMDB00574](http://hmdb.ca/scripts/show_card.cgi?METABOCARD=HMDB00574.txt) | 2 |
| 151 | unknown 60 |  | 2 |
| 152 | unknown 61 |  | 2 |
| 153 | 1-Aminocyclopentanecarboxylic acid |  | 2 |
| 154 | 5 Oxo-proline | [HMDB00267](http://www.hmdb.ca/scripts/show_card.cgi?METABOCARD=HMDB00267) | 2 |
| 155 | 1-Aminocyclopentanecarboxylic acid |  | 2 |
| 156 | 4-Hydroxyproline | [HMDB00725](http://www.hmdb.ca/scripts/show_card.cgi?METABOCARD=HMDB00725) | 2 |
| 157 | Creatine | [HMDB00064](http://www.hmdb.ca/scripts/show_card.cgi?METABOCARD=HMDB00064) | 2 |
| 158 | Leucine & Isoleucine (not resolved) | [HMDB00172 or HMDB00687](http://hmdb.ca/scripts/show_card.cgi?METABOCARD=HMDB00687.txt) | 2 |
| 159 | Homocysteine | [HMDB00742](http://www.hmdb.ca/scripts/show_card.cgi?METABOCARD=HMDB00742) | 2 |
| 160 | unknown 62 |  | 2 |
| 161 | Allysine | [HMDB01263](http://www.hmdb.ca/scripts/show_card.cgi?METABOCARD=HMDB01263) | 2 |
| 162 | Glutamate | [HMDB03339](http://hmdb.ca/scripts/show_card.cgi?METABOCARD=HMDB03339.txt) | 2 |
| 163 | Tryptophan | [HMDB00929](http://www.hmdb.ca/scripts/show_card.cgi?METABOCARD=HMDB00929) | 2 |
| 164 | Methionine | [HMDB00696](http://hmdb.ca/scripts/show_card.cgi?METABOCARD=HMDB00696.txt) | 2 |
| 165 | unknown 63 |  | 2 |
| 166 | unknown 64 |  | 2 |
| 167 | Carnitine | [HMDB00062](http://www.hmdb.ca/scripts/show_card.cgi?METABOCARD=HMDB00062) | 2 |
| 168 | Phenylalanine | [HMDB00159](http://www.hmdb.ca/scripts/show_card.cgi?METABOCARD=HMDB00159) | 2 |
| 169 | N-methylhistidine | [HMDB00479](http://www.hmdb.ca/scripts/show_card.cgi?METABOCARD=HMDB00479) | 2 |
| 170 | 5-Oxoproline | [HMDB00267](http://www.hmdb.ca/scripts/show_card.cgi?METABOCARD=HMDB00267) | 2 |
| 171 | Phenylalanine | [HMDB00159](http://www.hmdb.ca/scripts/show_card.cgi?METABOCARD=HMDB00159) | 2 |
| 172 | Leucine | [HMDB00687](http://www.hmdb.ca/scripts/show_card.cgi?METABOCARD=HMDB00687) | 2 |
| 173 | Unknown 65 |  | 2 |
| 174 | Citrulline | [HMDB00904](http://www.hmdb.ca/scripts/show_card.cgi?METABOCARD=HMDB00904) | 2 |
| 175 | unknown 66 |  | 2 |
| 176 | Cysteinylglycine | [HMDB00078](http://www.hmdb.ca/scripts/show_card.cgi?METABOCARD=HMDB00078) | 2 |
| 177 | Hippuric acid | [HMDB00714](http://www.hmdb.ca/scripts/show_card.cgi?METABOCARD=HMDB00714) | 2 |
| 178 | Tyrosine | [HMDB00158](http://www.hmdb.ca/scripts/show_card.cgi?METABOCARD=HMDB00158) | 2 |
| 179 | Aspartic acid | [HMDB06483](http://www.hmdb.ca/scripts/show_card.cgi?METABOCARD=HMDB06483) | 2 |
| 180 | unknown 67 |  | 2 |
| 181 | unknown 68 |  | 2 |
| 182 | unknown 69 |  | 2 |
| 183 | unknown 70 |  | 2 |
| 184 | unknown 71 |  | 2 |
| 185 | Glutamic acid | [HMDB00148](http://www.hmdb.ca/scripts/show_card.cgi?METABOCARD=HMDB00148) | 2 |
| 186 | Tryptophan | [HMDB00929](http://www.hmdb.ca/scripts/show_card.cgi?METABOCARD=HMDB00929) | 2 |
| 187 | unknown 72 |  | 2 |
| 188 | unknown 73 |  | 2 |
| 189 | unknown 74 |  | 2 |
| 190 | unknown 75 |  | 2 |
| 191 | 2-aminoadipate | [HMDB00510](http://www.hmdb.ca/scripts/show_card.cgi?METABOCARD=HMDB00510) | 2 |
| 192 | unknown 76 |  | 2 |
| 193 | unknown 77 |  | 2 |
| 194 | unknown 78 |  | 2 |
| 195 | unknown 79 |  | 2 |
| 196 | unknown 80 |  | 2 |
| 197 | unknown 81 |  | 2 |
| 198 | unknown 82 |  | 2 |
| 199 | Citrate | [HMDB00094](http://www.hmdb.ca/scripts/show_card.cgi?METABOCARD=HMDB00094) | 2 |
| 200 | Alanine + glucose (this is a byproduct) | [HMDB00161](http://hmdb.ca/scripts/show_card.cgi?METABOCARD=HMDB00161.txt) | 2 |
| 201 | glucose and/or other hexose -2H | [HMDB00122](http://hmdb.ca/scripts/show_card.cgi?METABOCARD=HMDB00122.txt) | 2 |
| 202 | glucose and/or other hexose | [HMDB00122](http://hmdb.ca/scripts/show_card.cgi?METABOCARD=HMDB00122.txt) | 2 |
| 203 | glucose and/or other hexose | [HMDB00122](http://hmdb.ca/scripts/show_card.cgi?METABOCARD=HMDB00122.txt) | 2 |
| 204 | glucose and/or other hexose | [HMDB00122](http://hmdb.ca/scripts/show_card.cgi?METABOCARD=HMDB00122.txt) | 2 |
| 205 | glucose and/or other hexose | [HMDB00122](http://hmdb.ca/scripts/show_card.cgi?METABOCARD=HMDB00122.txt) | 2 |
| 206 | unknown 83 |  | 2 |
| 207 | Citrate + NH4 | [HMDB00094](http://www.hmdb.ca/scripts/show_card.cgi?METABOCARD=HMDB00094) | 2 |
| 208 | Citrate + Na | [HMDB00094](http://www.hmdb.ca/scripts/show_card.cgi?METABOCARD=HMDB00094) | 2 |
| 209 | unknown 84 |  | 2 |
| 210 | unknown 85 |  | 2 |
| 211 | unknown 86 |  | 2 |
| 212 | unknown 87 |  | 2 |
| 213 | unknown 88 |  | 2 |
| 214 | unknown 89 |  | 2 |
| 215 | unknown 90 |  | 2 |
| 216 | unknown 91 |  | 2 |
| 217 | unknown 92 |  | 2 |
| 218 | unknown 93 |  | 2 |
| 219 | unknown 94 |  | 2 |
| 220 | unknown 95 |  | 2 |
| 221 | unknown 96 |  | 2 |
| 222 | unknown 97 |  | 2 |
| 223 | unknown 98 |  | 2 |
| 224 | unknown 99 |  | 2 |
| 225 | unknown 100 |  | 2 |
| 226 | unknown 101 |  | 2 |
| 227 | unknown 102 |  | 2 |
| 228 | unknown 103 |  | 2 |
| 229 | unknown 104 |  | 2 |
| 230 | unknown 105 |  | 2 |
| 231 | N-acetylglucosamine | [HMDB00215](http://hmdb.ca/scripts/show_card.cgi?METABOCARD=HMDB00215.txt) | 2 |
| 232 | unknown 106 |  | 2 |
| 233 | unknown 107 |  | 2 |
| 234 | unknown 108 |  | 2 |
| 235 | unknown 109 |  | 2 |
| 236 | unknown 110 |  | 2 |
| 237 | unknown 111 |  | 2 |
| 238 | unknown 112 |  | 2 |
| 239 | unknown 113 |  | 2 |
| 240 | unknown 114 |  | 2 |
| 241 | Glutathione | [HMDB00125](http://www.hmdb.ca/scripts/show_card.cgi?METABOCARD=HMDB00125) | 2 |
| 242 | unknown 115 |  | 2 |
| 243 | N-acetylneuraminate | [HMDB00230](http://hmdb.ca/scripts/show_card.cgi?METABOCARD=HMDB00230.txt) | 2 |
| 244 | unknown 116 |  | 2 |
| 245 | Tryptophan + glucose (this is a byproduct) | [HMDB00929](http://www.hmdb.ca/scripts/show_card.cgi?METABOCARD=HMDB00929) | 2 |
| 246 | unknown 117 |  | 2 |
| 247 | unknown 118 |  | 2 |
| 248 | unknown 119 |  | 2 |
| 249 | unknown 120 |  | 2 |
| 250 | unknown 121 |  | 2 |
| 251 | unknown 122 |  | 2 |
| 252 | unknown 123 |  | 2 |
| 253 | unknown 124 |  | 2 |
| 254 | unknown 125 |  | 2 |
| 255 | unknown 126 |  | 2 |
| 256 | unknown 127 |  | 2 |
| 257 | unknown 128 |  | 2 |
| 258 | unknown 129 |  | 2 |
| 259 | unknown 130 |  | 2 |
| 260 | unknown 131 |  | 2 |
| 261 | unknown 132 |  | 2 |
| 262 | unknown 133 |  | 2 |
| 263 | unknown 134 |  | 2 |
| 264 | unknown 135 |  | 2 |
| 265 | unknown 136 |  | 2 |
| 266 | unknown 137 |  | 2 |
| 267 | unknown 138 |  | 2 |
| 268 | unknown 139 |  | 2 |
| 269 | C16:0_LPC |  | 3 |
| 270 | C16:1_LPC |  | 3 |
| 271 | C18:0_LPC |  | 3 |
| 272 | C18:1_LPC |  | 3 |
| 273 | C18:2_LPC |  | 3 |
| 274 | C20:4_LPC |  | 3 |
| 275 | C22:6_LPC |  | 3 |
| 276 | C32:0_PC |  | 3 |
| 277 | C32:1_PC |  | 3 |
| 278 | C34:1_PC |  | 3 |
| 279 | C34:2_PC |  | 3 |
| 280 | C34:3_PC |  | 3 |
| 281 | C36:1_PC |  | 3 |
| 282 | C36:2_PC |  | 3 |
| 283 | C36:3_PC |  | 3 |
| 284 | C36:4_PC |  | 3 |
| 285 | C36:5_PC |  | 3 |
| 286 | C38:4_PC |  | 3 |
| 287 | C38:5_PC |  | 3 |
| 288 | C14:0_SPM |  | 3 |
| 289 | C15:0_SPM |  | 3 |
| 290 | C16:0_SPM |  | 3 |
| 291 | C16:1_SPM |  | 3 |
| 292 | C18:0_SPM |  | 3 |
| 293 | C22:0_SPM |  | 3 |
| 294 | C23:0_SPM |  | 3 |
| 295 | C23:1_SPM |  | 3 |
| 296 | C24:0_SPM |  | 3 |
| 297 | C24:1_SPM |  | 3 |
| 298 | C16:0_ChE |  | 3 |
| 299 | C16:1_ChE |  | 3 |
| 300 | C18:1_ChE |  | 3 |
| 301 | C18:2_ChE |  | 3 |
| 302 | C18:3_ChE |  | 3 |
| 303 | C20:4_ChE |  | 3 |
| 304 | C20:5_ChE |  | 3 |
| 305 | C22:6_ChE |  | 3 |
| 306 | C44:0_TG |  | 3 |
| 307 | C44:1_TG |  | 3 |
| 308 | C46:0_TG |  | 3 |
| 309 | C46:1_TG |  | 3 |
| 310 | C46:2_TG |  | 3 |
| 311 | C48:0_TG |  | 3 |
| 312 | C48:1_TG |  | 3 |
| 313 | C48:2_TG |  | 3 |
| 314 | C50:1_TG |  | 3 |
| 315 | C50:2_TG |  | 3 |
| 316 | C52:2_TG |  | 3 |
| 317 | C54:2_TG |  | 3 |
| 318 | C48:3_TG |  | 3 |
| 319 | C50:3_TG |  | 3 |
| 320 | C50:4_TG |  | 3 |
| 321 | C52:3_TG |  | 3 |
| 322 | C52:4_TG |  | 3 |
| 323 | C52:5_TG |  | 3 |
| 324 | C54:3_TG |  | 3 |
| 325 | C54:4_TG |  | 3 |
| 326 | C54:5_TG |  | 3 |
| 327 | C54:6_TG |  | 3 |
| 328 | C56:5_TG |  | 3 |
| 329 | C56:6_TG |  | 3 |
| 330 | C14:0_FA |  | 4 |
| 331 | C16:0_FA |  | 4 |
| 332 | C16:1_FA |  | 4 |
| 333 | C18:0_FA |  | 4 |
| 334 | C18:1_FA |  | 4 |
| 335 | C18:2_FA |  | 4 |
| 336 | C18:3_FA |  | 4 |
| 337 | C20:0_FA |  | 4 |
| 338 | C20:1_FA |  | 4 |
| 339 | C20:2_FA |  | 4 |
| 340 | C20:3_FA |  | 4 |
| 341 | C20:4_FA |  | 4 |
| 342 | C22:5_FA |  | 4 |
| 343 | C22:6_FA |  | 4 |
